# The Effector RipAW Enhances *Ralstonia solanacearum* Invasion in *Arabidopsis* via CBP60g/SARD1‐Dependent and ‐Independent Pathways

**DOI:** 10.1111/mpp.70207

**Published:** 2026-01-21

**Authors:** Huijuan Wang, Shouyang Fu, Tao Cao, Yang Niu, Shengyang Cheng, Qichang Gong, Hui Ma, Xiang Wang, Jinxue Hu, Min Chen, Dongdong Wang, Yong Zhang, Nuria S. Coll, Marc Valls, Qin Chen, Cuizhu Zhao, Yue Chen, Haibin Lu

**Affiliations:** ^1^ State Key Laboratory for Crop Stress Resistance and High‐Efficiency Production, College of Agronomy Northwest A&F University Yangling China; ^2^ College of Resources and Environment Southwest University Chongqing China; ^3^ Interdisciplinary Research Center for Agriculture Green Development in Yangtze River Basin Southwest University Chongqing China; ^4^ Centre for Research in Agricultural Genomics (CSIC‐IRTA‐UAB‐UB) Bellaterra Catalonia Spain; ^5^ Consejo Superior de Investigaciones Científicas (CSIC) Barcelona Spain; ^6^ Department of Genetics, Microbiology and Statistics University of Barcelona Barcelona Catalonia Spain; ^7^ College of Food Science and Engineering Northwest A&F University Yangling China

**Keywords:** CBP60g, plant susceptibility, *Ralstonia solanacearum*, RipAW, SARD1

## Abstract

CaM‐binding Protein 60‐like G (CBP60g) and Systemic Acquired Resistance Deficient 1 (SARD1) are key immune signalling regulators that redundantly promote salicylic acid (SA) biosynthesis and plant immunity. Pathogen effectors often target these immune nodes to suppress plant defence. However, the role of bacterial effectors in disabling CBP60g and SARD1 to increase plant susceptibility remains unclear. In this study, we show that RipAW, an E3 ligase effector from *Ralstonia solanacearum* , induces root architecture changes and enhances plant susceptibility to *R. solanacearum* in *Est::RipAW* transgenic plants. The constitutively expressed *RipAW (C177S)*, lacking E3 ligase activity, did not affect root architecture or plant susceptibility, indicating that RipAW's E3 ligase activity is crucial for these phenotypes. Transcriptional profiling of *Est::RipAW* plants revealed strong up‐regulation of *CBP60g* and *SARD1,* while the SA signalling pathway remained in a basal state. Transient expression of *RipAW* and *CBP60g* in *Nicotiana benthamiana* showed that RipAW associates with CBP60g and affects its stability. Genetic analysis revealed that loss‐of‐function mutations in *CBP60g* and *SARD1* increased plant susceptibility to *R. solanacearum,* but did not enhance RipAW‐mediated pathogen growth. Furthermore, growth of the *R. solanacearum* Δ*RipAW* null mutant strain was reduced in wild‐type plants but restored in *cbp60g/sard1* mutant plants, confirming that the promotion of RipAW on bacterial growth is dependent on CBP60g and SARD1. Surprisingly, CBP60g and SARD1 were not involved in *R. solanacearum* ‐induced and RipAW‐triggered root architecture changes. Overall, our findings demonstrate that RipAW increases plant susceptibility to *R. solanacearum* via both CBP60g/SARD1‐dependent and ‐independent pathways.

## Graphical abstract

The effector RipAW facilitates *R. solanacearum* infection by suppressing CBP60g/SARD1‐dependent SA pathway and triggering CBP60g/SARD1‐independent root architecture changes.

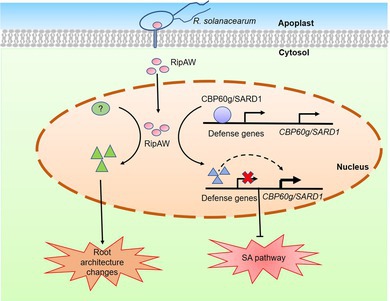

## 1 Introduction

*Ralstonia solanacearum* is one of the most destructive plant pathogens. It causes vascular bacterial wilt disease on many crops including the Solanaceae family, resulting in huge crop production losses and threatening human food security (Coll and Valls 2013). Successful *R. solanacearum* colonisation involves bacterial entry into the roots, movement from roots to shoots, circular vascular bundle invasion and radial apoplastic spread in the cortex (Genin and Denny 2012; Planas‐Marques et al. 2020). Genetic diversity confers *R. solanacearum* a wide host range and strong adaptation to diverse climates and environments, with the pathogen also being referred to as *R. solanacearum* species complex (RSSC) (Genin and Denny 2012). *R. solanacearum* employs various strategies to successfully thrive in plants, including production of exopolysaccharides, phytohormone biosynthesis and secretion of cell wall‐degrading enzymes to colonise plant tissues (Genin and Denny 2012). However, the main virulence determinant of *R. solanacearum* is its type III secretion system (T3SS), a conserved syringe‐like bacterial apparatus. The T3SS is responsible for translocation of type III effector proteins into the host cell. Inside the host cell, effectors manipulate physiological processes to suppress plant immunity and to make nutrients available in the apoplast for bacterial multiplication (Coll and Valls 2013).

Long‐term coevolution with phytopathogens has resulted in the acquisition of a sophisticated immune system in plants to fend off potential pathogens. Activation of the plant immune system relies on pathogen recognition via cell surface and intracellular immune receptors. Cell surface immune receptors include receptor‐like kinases (RLKs) and receptor‐like proteins (RLP), which directly perceive diverse apoplastic immunogenic molecular patterns derived from microbes or plant self, including conserved microbe‐associated molecular patterns (MAMPs), plant damage‐associated molecular patterns (DAMPs) and phytocytokines (DeFalco and Zipfel 2021; Zhou and Zhang 2020). The activation of cell‐surface receptors leads to multiple important downstream immune signalling events, such as calcium influx, reactive oxygen species (ROS) burst, activation of MAPK signalling cascade, callose deposition, production of phytocytokines and transcriptional reprogramming, together referred as pattern‐triggered immunity (PTI) (DeFalco and Zipfel 2021, Zhou and Zhang 2020). Intracellular immune receptors, belonging to the nucleotide‐binding site and leucine‐rich‐repeat (NLR) protein family, sense pathogen effectors secreted into the plant cell, resulting in triggering NLR resistosome formation and further activation of effector‐triggered plant immunity (ETI) (Bi and Zhou 2021; Duxbury et al. 2021). Both ETI and PTI signalling cascades share components and potentiate each other at the transcriptional, translational and post‐translational levels (Ngou et al. 2022).

Upon pathogen infection, the phytohormone salicylic acid (SA) is rapidly synthesised and accumulates, activating plant resistance. Pathogen‐induced SA accumulation mainly relies on the isochorismate synthase pathway (Peng et al. 2021). In *Arabidopsis thaliana* , Isochorismate Synthase 1 (ICS1) converts chorismate to isochorismate in chloroplasts (Peng et al. 2021). Isochorismate is then exported from plastids to the cytosol by the enhanced disease susceptibility 5 (EDS5) transporter, where an amidotransferase AvrPphB Susceptible 3 (PBS3) catalyses the conjugates of glutamate to isochorismate to produce isochorismate‐9‐glutamate (IC‐9‐Glu) (Rekhter et al. 2019). Finally, IC‐9‐Glu spontaneously decays into SA or is converted to SA by Enhanced Pseudomonas Susceptibility 1 (EPS1) (Rekhter et al. 2019; Torrens‐Spence et al. 2019). These SA biosynthesis‐related genes are strictly regulated to avoid autoimmunity triggered by overaccumulation of the hormone. CaM‐binding Protein 60‐like G (CBP60g) and SAR Deficient 1 (SARD1) are transcription factors essential for pathogen‐induced expression of SA biosynthetic genes such as *ICS1*, *EDS5* and *PBS3* (Sun et al. 2015). Simultaneous *CBP60g* and *SARD1* deficiency abolishes pathogen‐induced SA accumulation (Zhang, Xu, et al. 2010). CBP60g and SARD1 directly associate with promoter regions of SA biosynthetic genes to turn on their expression (Sun et al. 2015). In addition, they also target key signalling components of PTI and ETI and induce their expression (Sun et al. 2015). Unexpectedly, a number of negative regulators of plant immunity are also targets of CBP60g and SARD1 (Sun et al. 2018, 2015). Hence, CBP60g and SARD1 serve as master regulators in PTI, ETI, basal defence and systemic acquired resistance (SAR) (Peng et al. 2021; Sun et al. 2015).

Because of their central role in defence, CBP60g and SARD1 could constitute pathogen targets to modulate plant resistance. In fact, the *Verticillium dahliae* fungal effector VdSCP41 has been reported to reduce CBP60g and SARD1 transcriptional activity to suppress plant immunity (Qin et al. 2018). Given that CBP60g and SARD1 play a central role in plant immunity, it is highly likely that various effector proteins from different pathogens target these two proteins to manipulate the plant's immune responses. In this way, CBP60g and SARD1 could function as key hubs or nodes in the immune signalling network, similar to other proteins in plant immunity that have been shown to be targeted by effectors to disrupt defence mechanisms. This is the case, for example, of receptor‐like cytoplasmic kinase BIK1 that integrates signals from multiple cell surface receptors and transduces signals to downstream components including RBOHD, calcium channel CNGC2/CNGC4 and MAPK cascades (DeFalco and Zipfel 2021; Zhou and Zhang 2020). *Pseudomonas syringae* effector AvrPphB, *R. solanacearum* effector RipAC and *Xanthomonas campestris* effector AvrAC target BIK1 to suppress PTI responses via post‐translational modifications (Feng et al. 2012; Zhang, Li, et al. 2010).

Upon *R. solanacearum* infection, *A. thaliana* root architecture undergoes remarkable changes. These include root hair formation around the meristem, primary root growth retardation, massive cell death at the tip and lateral root emergence sites (Lu et al. 2018; Zhao et al. 2019). These root structural changes caused by *R. solanacearum* infection are dependent on the T3SS and type III effectors (Lu et al. 2018; Zhao et al. 2019). Recently, the *R. solanacearum* effector RipAC has been reported to trigger lateral root formation and enhance pathogen infection (Yu et al. 2024). However, deletion of *RipAC* only partially blocked *R. solanacearum* ‐triggered lateral root formation (Yu et al. 2024), suggesting that other effectors may also be responsible for lateral root formation. Moreover, it is not clear which effector affects other root architecture changes such as cell death, root hair formation and primary root length retardation. The RipAW effector was previously reported to suppress PTI by promoting degradation of FLS2 and BIK1 in *Arabidopsis* (Sun et al. 2023). Here, we report that transient expression of *RipAW* inhibits primary root elongation, promotes root hair and lateral root formation, and enhances plant susceptibility to *R. solanacearum* . Global transcriptome profiling reveals that expression of *CBP60g* and *SARD1* is up‐regulated by *RipAW* transient expression. Simultaneous mutations of *CBP60g* and *SARD1* accelerate wilting disease symptoms and promote *R. solanacearum* proliferation. When co‐expressing RipAW with CBP60g in *N. benthamiana*, RipAW associates with them and degrades them through the 26S proteasome‐dependent pathway. However, CBP60g and SARD1 are not required for RipAW‐induced root architecture changes. Taken together, our data indicate that the *R. solanacearum* effector RipAW promotes *R. solanacearum* colonisation by destabilising CBP60g and SARD1 and induces root architecture changes via a different pathway, yet to be identified.

## 2 Results

### 2.1 Type III Effector RipAW From *R. solanacearum* GMI1000 Increases Virulence in *Arabidopsis*

**FIGURE 1:**
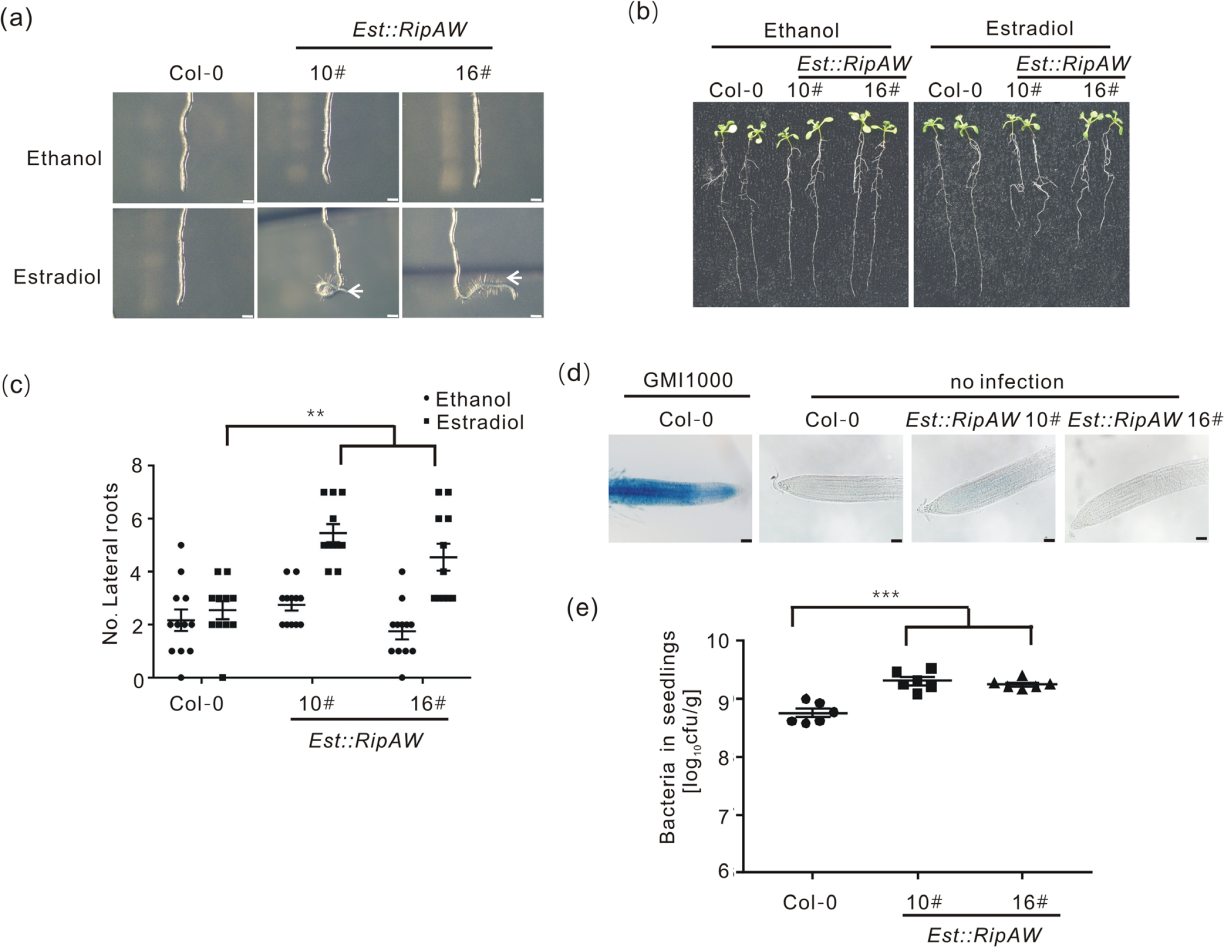
The expression of *RipAW* induces root architecture changes and increases *Ralstonia solanacearum* proliferation in seedlings. Six‐day‐old *Est::RipAW* seedlings were transferred onto MS− medium containing 5 μM β‐estradiol for inducing *RipAW* expression. (a) Representative pictures of root curvature and root hair phenotypes of *Est::RipAW* transgenic lines (10# and 16#) 2 days after β‐estradiol treatment (dpe) (*n* = 10, bar = 1 mm). Root hairs were photographed with an Olympus microscope. Arrow indicates root hairs. (b) Root growth of *Est::RipAW* transgenic lines at 5 dpe (*n* = 10). (c) Number of lateral roots of *Est::RipAW* lines at 5 dpe (*n* = 11). (d) Cell death on root tips of *Est::RipAW* lines. Cell death on infected seedlings was observed after Evans blue staining and photographed with an Olympus BX53 microscope at 2 dpe (*n* = 10, bar = 50 μm). (e) Bacterial growth in *Est::RipAW* transgenic lines. One‐week‐old seedlings pretreated with 5 μM β‐estradiol were inoculated with a *R. solanacearum* suspension (OD_600_ = 0.0001). Bacterial content in seedlings was measured at 3 days post‐inoculation (*n* = 12). All experiments were repeated at least three times with similar results. Two‐way ANOVA Dunnett's test (**p < 0.01) was used in (c) and one‐way ANOVA Dunnett's test (***p < 0.001) in (e). Values in figures represent mean ± SE.

The *R. solanacearum* effector RipAW is perceived by different *Nicotiana* species and triggers plant immunity (Niu et al. 2022). To investigate the mechanism whereby RipAW enhances *R. solanacearum* virulence, we first tried to obtain transgenic *Arabidopsis* plants constitutively expressing *RipAW* under a CaMV 35S promoter control. Transgenic plants expressing *RipAW* (*35S::RipAW*, T_1_ generation) displayed a dwarf phenotype and did not produce seeds (Figure S1a), suggesting that *RipAW* constitutive overexpression is detrimental for plant growth and development. Therefore, we generated transgenic *Arabidopsis* plants conditionally expressing *RipAW* under the control of a β‐estradiol‐inducible promoter (*Est::RipAW*) (Figure S1b). Considering that infection with *R. solanacearum* alters *Arabidopsis* seedling root architecture (Lu et al. 2018), we investigated whether the expression of *RipAW* could mimic this phenotype in transgenic seedlings. The *Est::RipAW* seedlings exhibited shorter and strongly curved roots with altered gravitropism compared to wild‐type (WT) seedlings (Figure 1a,b). Moreover, the *Est::RipAW* seedlings also displayed many more root hairs and lateral roots than WT seedlings (Figure 1a,c), reminiscent of root alterations induced by *R. solanacearum* (Lu et al. 2018). However, *Est::RipAW* roots did not show cell death in the absence of pathogen inoculation (Figure 1d). Together, these data indicate that RipAW may be involved in all *R. solanacearum* ‐induced root architecture changes except cell death.

Expression of *RipAW* alone was sufficient to cause *R. solanacearum* ‐triggered root structural changes at early stages of the infection process (Lu et al. 2018; Zhao et al. 2019). We guessed it may contribute to *R. solanacearum* colonisation in roots. Therefore, a gnotobiotic root infection system for evaluating plant resistance to *R. solanacearum* was established, which also facilitated estradiol application. To understand if RipAW promotes *R. solanacearum* proliferation in plants, we inoculated WT and *Est::RipAW* transgenic seedlings pretreated by β‐estradiol with the *R. solanacearum* GMI1000 strain and measured pathogen colonisation. Bacterial populations in *Est::RipAW* seedlings were significantly higher than those in WT seedlings (Figure 1e), suggesting that RipAW increases plant susceptibility to *R. solanacearum* .

### 2.2 Deletion of *RipAW* Reduces *R. solanacearum* Virulence in *Arabidopsis*

**FIGURE 2:**
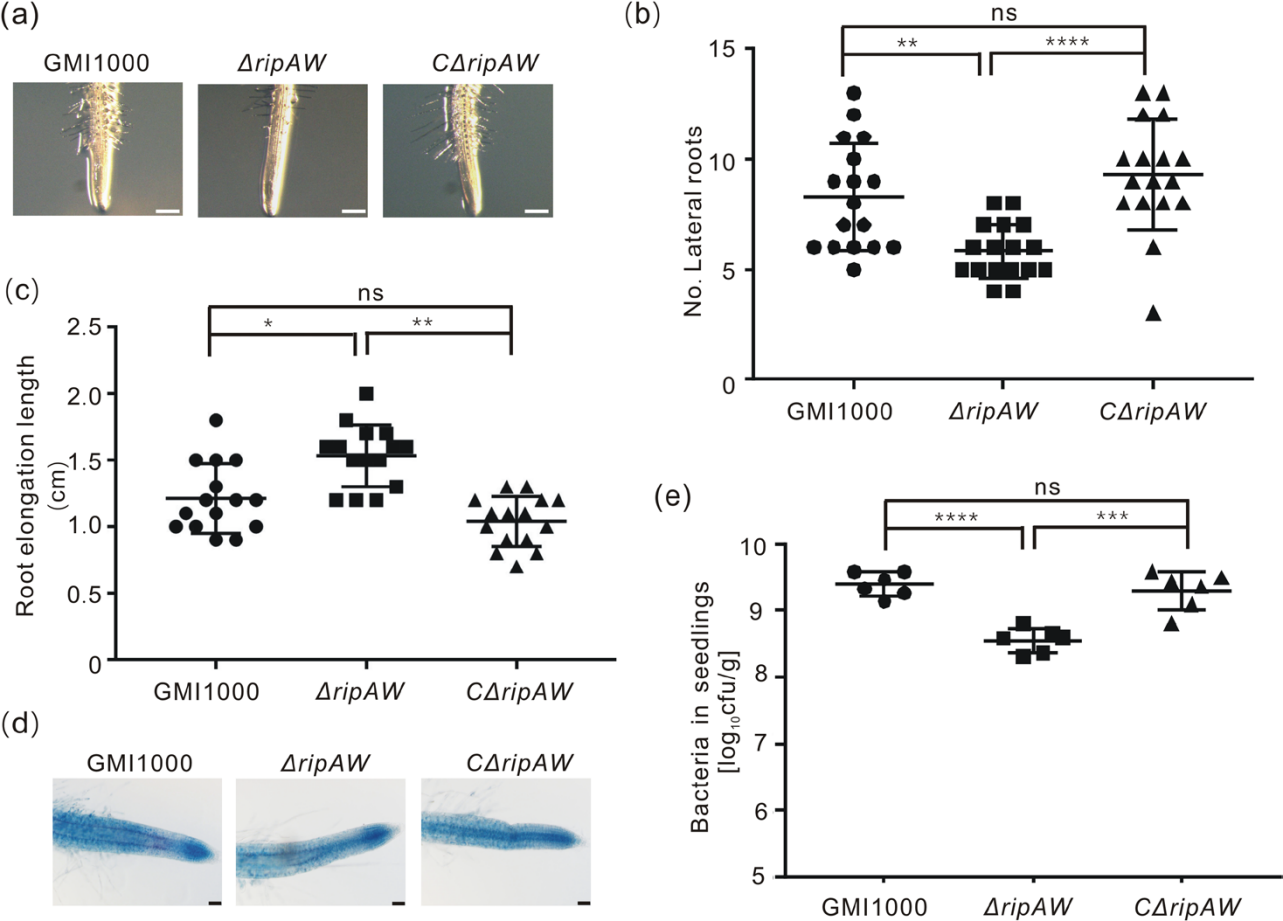
The effector RipAW is essential for *Ralstonia solanacearum* ‐induced root structure changes and the pathogen colonisation in plants. Six‐day‐old *Arabidopsis* seedlings grown on MS− medium were inoculated with either *R. solanacearum* GMI1000 wild type (WT), a RipAW mutant (Δ*ripAW*) or a complemented Δ*ripAW* mutant (*C*Δ*ripAW*). (a) Root hair formation triggered by pathogens at 1 day post‐inoculation (dpi) (*n* = 10, bar = 200 μm). (b) Lateral root formation. The number of lateral roots was counted at 4 dpi (*n* = 16). (c) Suppression of primary root growth. Root elongation was measured at 4 dpi (*n* = 16). (d) Cell death. Root tips were stained with Evans blue solution to visualise cell death at 2 dpi. Representative pictures obtained with an Olympus microscope are shown (*n* = 8, bar = 50 μm). (e) Bacterial populations in seedlings at 4 dpi (*n* = 12). All experiments were repeated at least twice with similar results. Statistical analysis was performed using the one‐way ANOVA Tukey's test (*p < 0.05, **p < 0.01, ***p < 0.001 and ****p < 0.0001). Values in figures represent mean ± SE.

To further ascertain the importance of RipAW for *R. solanacearum* infection on plants, we generated a *RipAW* deletion mutant (Δ*ripAW*) and its complemented strain (*C*Δ*ripAW*) in the *R. solanacearum* GMI1000 background (Niu et al. 2022) (Figure S2a–c) and investigated their virulence on *Arabidopsis*. The seedlings infected with the mutant strain displayed fewer root hairs and lateral roots, but significantly longer roots compared to those seedlings infected with the WT strain or the complemented strain *C*Δ*ripAW* (Figure 2a–c). However, the seedlings inoculated with the mutant strain still displayed cell death on the root tip, which is comparable to that caused by the WT and the complemented strains (Figure 2d). Next, we measured growth of the mutant, WT and complemented strains in *Arabidopsis* seedlings in vitro. The growth of Δ*ripAW* in seedlings was significantly lower than that of WT and the complemented strains (Figure 2e). The growth defects of Δ*ripAW* observed in *Arabidopsis* seedlings was further confirmed in adult plants using a hydroponic infection system (Figure S2d). Altogether, our data indicate that RipAW is required for *R. solanacearum* virulence in plants.

### 2.3 E3 Ligase Activity Is Essential for the RipAW Virulence Function in Plants

**FIGURE 3:**
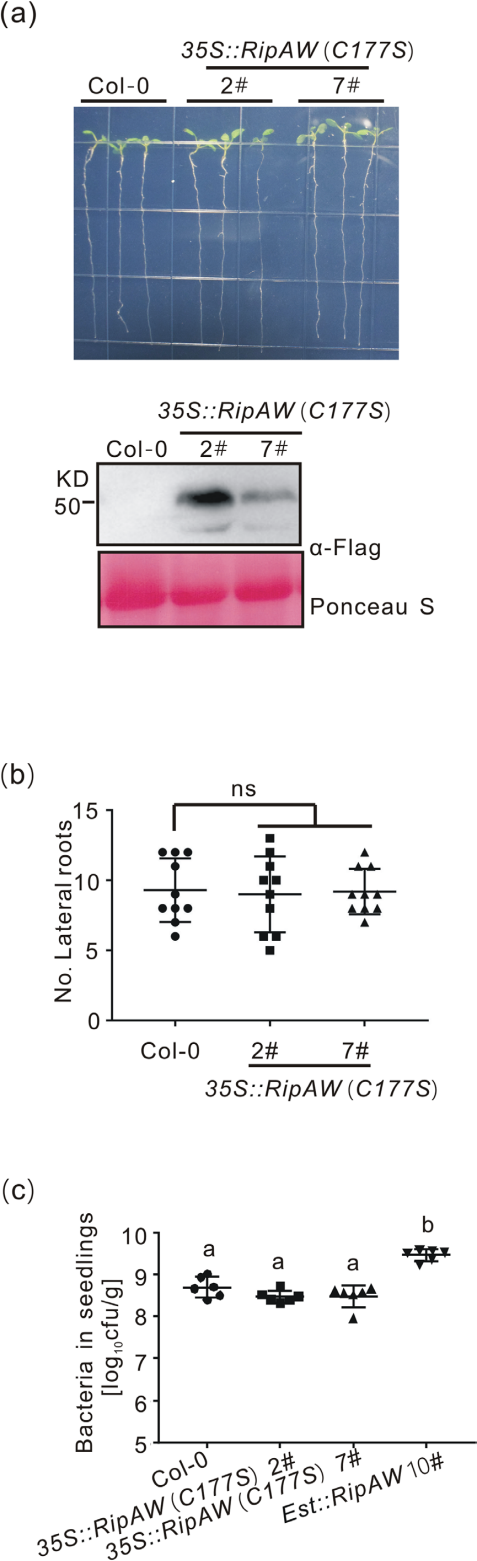
E3 ligase activity is required for RipAW‐triggered root phenotypes and bacterial growth. (a) RipAW (C177S) did not affect root architecture. Root pictures were taken at 11 days after sowing the seeds (upper panel, *n* = 10) and RipAW (C177S) protein in plants was detected by western blot (lower panel). (b) Number of lateral roots of plants expressing *RipAW (C177S)* at 16 days after sowing the seeds (*n* = 10). (c) Bacterial population in *RipAW (C177S)* seedlings. Six‐day‐old seedlings were inoculated with a *R. solanacearum* GMI1000 suspension (OD_600_ = 0.0001). Bacterial populations in seedlings was measured at 3 days post‐inoculation (*n* = 12). The experiments were repeated three times with similar results. Statistical analysis was performed using one‐way ANOVA Dunnett's test (b; ns, no significant difference) and one‐way ANOVA Tukey's test (c, letters on columns indicate statistically significant differences). Values in figures represent mean ± SE.

RipAW is a functional E3 ligase, with its activity playing a critical role in RipAW‐triggered cell death in *Nicotiana benthamiana* (Niu et al. 2022). The cysteine residue at the 177 position is required for its E3 ligase activity (Niu et al. 2022). To explore the importance of E3 ligase activity for RipAW virulence in *Arabidopsis*, we generated transgenic plants constitutively expressing *RipAW (C177S)* driven by a CaMV 35S promoter (*35S::RipAW (C177S)*). The transgenic lines showed similar root length as WT plants (Figure 3a) and no significant differences in lateral root number were observed (Figure 3b), indicating that the cysteine residue is required for RipAW‐induced root architecture changes. We then inoculated seedlings of WT and two *RipAW (C177S)* transgenic lines with *R. solanacearum* in vitro. At 4 days post‐inoculation (dpi), bacterial loads in the *RipAW (C177S)* transgenic lines were indistinguishable from those in WT seedlings and lower than in the *Est::RipAW* transgenic plants (Figure 3c), proving that the E3 ligase activity of RipAW is essential to facilitate *R. solanacearum* proliferation in plants.

### 2.4 Transcriptome Profiling Reveals That CBP60g and SARD1 Upregulation in *Est::RipAW* Transgenic Plants Does Not Activate the SA Signalling Pathway

**FIGURE 4:**
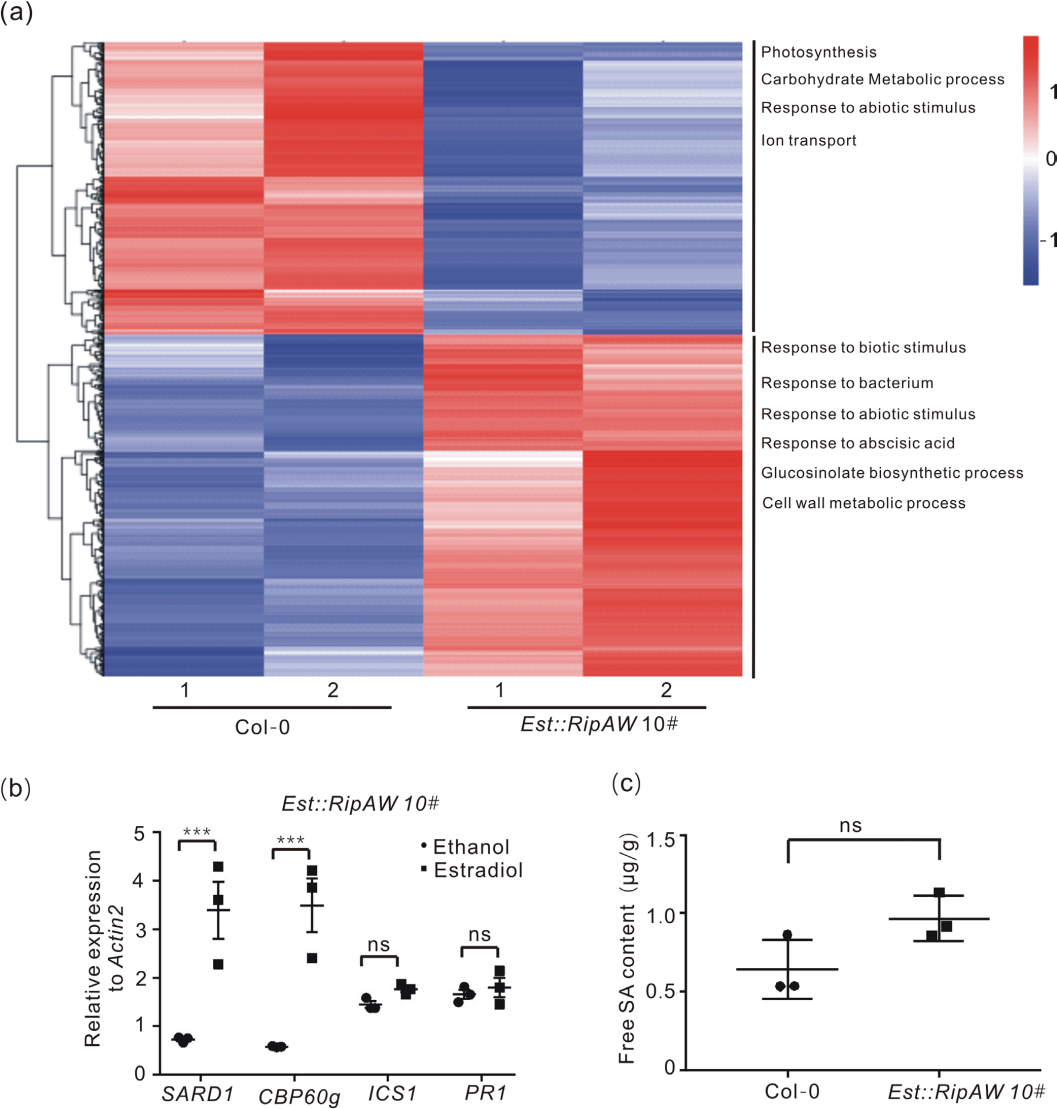
The salicylic acid (SA) signalling pathway is inhibited in *Est::RipAW* transgenic plants. (a) Cluster analysis of the RNA‐seq data. The heatmap displays the expression patterns of 993 differentially expressed genes in roots of *Est::RipAW* transgenic plants. The left vertical axis represents groups of co‐expressed genes while the horizontal axis displays sample names. The top over‐represented gene ontology (GO) terms in up‐regulated DEGs and down‐regulated DEGs are shown on the right. The heatmap depicts the FPKM (fragments per kilobase per million reads) values after log_10_ transformation. (b) Expression of *CBP60g* and *SARD1*, but not *ICS1* and *PR1*, was up‐regulated in response to *RipAW* expression. Gene expression of *CBP60g*, *SARD1*, *ICS1* and *PR1* was measured by reverse transcription‐quantitative PCR at 48 h post‐β‐estradiol treatment with gene‐specific primers. Gene expression was quantified and normalised to *AtActin2* using the 2^−ΔΔ*C*t^ method. The experiment was performed three times with similar results. Statistical analysis was performed by two‐way ANOVA Sidak's test (***p < 0.001, ns, no significant difference). (c) SA content in the roots of *Est::RipAW* transgenic line. Five‐week‐old plants grown in *Arabidopsis* nutrient solution (ANS) were transferred into ANS supplemented with 25 μM β‐estradiol. 48 h later, roots were collected and SA content in samples was measured by UPLC‐ESI‐MS/MS. Statistical analysis was performed by Student's *t* tests (ns, no significant difference).

To determine the mechanism(s) whereby RipAW employs to alter plant defence, we analysed RipAW‐mediated transcriptional reprogramming using only one *Est::RipAW* line because both lines exhibited very similar phenotypes (Figure 1). For this, total RNA was extracted from roots of 6‐day‐old *Est::RipAW* line 10# and WT seedlings after β‐estradiol treatment and subjected to Illumina deep sequencing. A total of 993 *Arabidopsis* differentially expressed genes (DEGs) were identified in *Est::RipAW* transgenic plants compared to WT (Figure 4a). 534 genes exhibited up‐regulated expression patterns while 459 genes were down‐regulated by RipAW (Figure 4a, Data S1).

To identify the signalling pathways or physiological processes altered by RipAW in the plant, we analysed these DEGs with enrichment of gene ontology (GO) terms. The down‐regulated genes were mainly enriched in energy production‐related and abiotic stress GO terms such as ‘photosynthesis’, ‘carbohydrate metabolic process’, ‘ion transport’ and ‘response to abiotic stress’, but no GOs related to defence were identified (Figure 4a). On the contrary, defence‐related GO terms such as ‘response to biotic stress’, ‘response to bacterium’ and ‘glucosinolate biosynthetic process’ were over‐represented among the up‐regulated genes. Additionally, genes related to the GO terms ‘response to abscisic acid’ and ‘cell wall metabolic process’ were also up‐regulated (Figure 4a).

Among these DEGs, we noticed that *CBP60g* and *SARD1*, two critical transcription activators of plant immunity (Sun et al. 2015), were significantly up‐regulated in the *Est::RipAW* transgenic plants (Table 1 and Table S2). The transcriptional activation of *CBP60g* and *SARD1* was further confirmed in two *Est:RipAW* transgenic lines (10# and 16#) by reverse transcription‐quantitative PCR (RT‐qPCR) (Figure 4b and Figure S3). Upon pathogen infection, CBP60g and SARD1 are recruited to the promoter regions of *ICS1* and turn on its expression, leading to SA accumulation and activation of SA‐dependent plant resistance (Zhang, Xu, et al. 2010). The up‐regulation of *CBP60g* and *SARD1* prompted us to speculate whether the expression of genes involved in SA biosynthesis and signalling pathways was also turned on. Unexpectedly, our RNA‐seq data showed that the expression levels of 19 well‐known genes involved in SA biosynthesis and signalling pathways, except CBP60g and SARD1, remained unchanged in *Est::RipAW* transgenic lines (Table 1 and Table S2). To confirm this, we measured the RNA expression levels of the SA biosynthesis gene *ICS1* and the SA downstream marker gene *PR1* in *Est::RipAW* plants and WT plants. The results showed that the expressions of *ICSI* and *PR1* were not affected by RipAW (Figure 4b). We further measured endogenous free SA content and noticed that the SA content in *Est::RipAW* transgenic plants was indistinguishable from that in WT plants (Figure 4c), indicating that the up‐regulation of *CBP60g* and *SARD1* did not induce a strong SA accumulation in roots. Altogether, these results indicated that the increase of *CBP60g* and *SARD1* transcripts in *Est::RipAW* plants does not lead to SA accumulation and SA‐dependent immunity activation.

**TABLE 1:** RNA‐seq data indicated that nearly all of the genes involved in salicylic acid (SA) biosynthesis and signalling were not affected by RipAW expression.

Gene ID^a^	Gene name	Log_2_FC(RipAW/WT)	*p*	*p*_adj_ ^b^
AT1G74710	*ICS1*	0.202	0.511	0.934
AT4G39030	*EDS5*	0.703	0.006	0.096
AT5G13320	*PBS3*	3.661	0.004	0.078
AT5G67160	*EPS1*	0.086	0.787	0.980
AT3G09830	*PCRK1*	−0.032	0.894	0.991
AT5G03320	*PCRK2*	−0.044	0.846	0.986
AT2G22300	*CAMTA3*	−0.030	0.920	0.993
AT3G48090	*EDS1*	0.208	0.265	0.806
AT3G52430	*PAD4*	0.340	0.126	0.613
AT1G64280	*NPR1*	−0.369	0.080	0.504
AT5G45110	*NPR3*	0.900	8.9e−06	5.7e−04
AT4G19660	*NPR4*	−0.029	0.872	0.988
AT5G65210	*TGA1*	−0.055	0.715	0.976
AT5G06950	*TGA2*	−0.136	0.563	0.949
AT1G22070	*TGA3*	−0.081	0.844	0.985
AT5G10030	*TGA4*	0.222	0.280	0.819
AT5G06960	*TGA5*	−0.306	0.105	0.566
AT3G12250	*TGA6*	−0.256	0.331	0.850
AT1G77920	*TGA7*	0.535	0.032	0.307
AT2G13810	*ALD1*	−1.323	0.034	0.324
AT5G52810	*SARD4*	−0.001	0.995	0.999
AT1G19250	*FMO1*	1.969	0.132	0.626
AT1G73805	*SARD1*	3.640	3.9e−05	0.002
AT5G26920	*CBP60g*	2.550	8.2e−04	0.023

^a^ The genes presented here were all of reported genes involved in SA biosynthesis and signalling detected by RNA‐seq.

^b^ Adjusted *p* value.

### 2.5 RipAW Associates With and Destabilises CBP60g in E3 Ligase Activity‐Dependent Way

**FIGURE 5:**
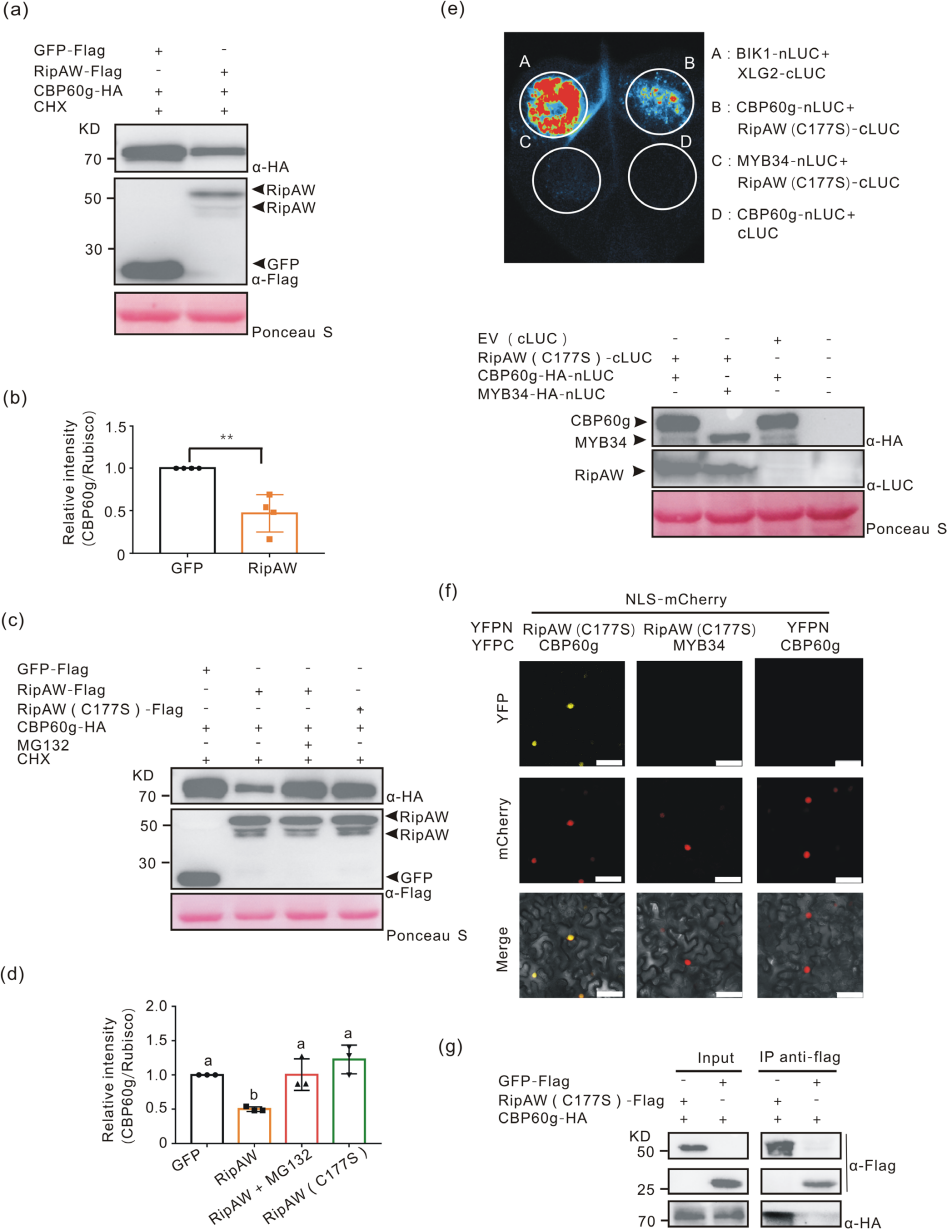
RipAW associates with and affects the stability of CBP60g. (a) RipAW destabilises CBP60g. Leaves of *Nicotiana benthamiana* were agro‐infiltrated with *RipAW* and *CBP60g*. At 1 day post‐infiltration (dpi), total proteins of all samples were extracted after cycloheximide (CHX) treatment and western blot was performed with anti‐FLAG and anti‐HA antibodies. (b) Quantification of signal intensity of CBP60g relative to RuBisCO in (a). Signal intensity of different samples was analysed using ImageJ software. Statistical analysis was performed by Student's *t* test (two‐tailed, **p < 0.01). (c) The degradation of RipAW on CBP60g is E3 ligase activity‐dependent. RipAW and its E3 ligase mutant RipAW (C177S) were co‐expressed with CBP60g in *N. benthamiana* in the absence or presence of the 26S proteasome inhibitor MG132. At 1 dpi, leaves were treated with CHX for 3 h. Total proteins were extracted and immunoblot was performed with anti‐FLAG and anti‐HA antibody. (d) Quantification of signal intensity of CBP60g relative to RuBisCO in (c). Statistical analysis was done by one‐way ANOVA Tukey's test (letters on columns indicate statistically significant differences). (e) RipAW (C177S) associates with CBP60g, indicated by split‐luciferase assay. Leaves of *N. benthamiana* were infiltrated with agrobacteria carrying the indicated genes. Luciferase activity imaging of agro‐infiltrated leaves was obtained using an ultrasensitive CCD camera at 2 dpi. (f) Bimolecular fluorescence complementation shows that RipAW (C177S) interacts with CBP60g in the nucleus. mCherry carrying an nuclear localisation signal (NLS) was used to highlight nuclei. The fluorescence signal of leaves expressing the indicated genes was detected by a laser‐scanning confocal microscope (Zeiss; LSM880) at 2 dpi, bar = 50 μm. (g) CBP60g co‐immunoprecipitates with RipAW (C177S). Total proteins from *N. benthamiana* leaves agro‐infiltrated with the indicated genes were extracted and co‐immunoprecipitation (CoIP) assay was performed at 2 dpi. Proteins were separated on an SDS‐PAGE and detected with anti‐FLAG and anti‐HA antibodies. All the experiments were repeated more than three times with similar results.

Because RipAW is a functional E3 ligase, we hypothesised that it might affect the stability of CBP60g and SARD1. Hence, we tested whether the protein levels of CBP60g and SARD1 changed in the presence of RipAW. To this end, we investigated the stability of CBP60g by co‐expressing RipAW and CBP60g in *N. benthamiana*. To decrease the effect of protein biosynthesis on CBP60g proteins, we temporarily blocked protein biosynthesis using the protein biosynthesis inhibitor cycloheximide (CHX). Compared to GFP, the protein levels of CBP60g were significantly reduced when co‐expressed with *RipAW* while the chimeric protein GFP‐HA‐nLUC was not affected by RipAW (Figure 5a,b and Figure S4a,b). To determine whether the RipAW E3 ligase activity is important for its effect on CBP60g stability, we co‐infiltrated *RipAW (C177S)* and *CBP60g* into *N. benthamiana* and observed that the C177S mutation abolishing E3 ligase activity did not result in CBP60g degradation (Figure 5c,d). Furthermore, in the presence of MG132, an inhibitor of ubiquitin‐mediated protein degradation, RipAW‐mediated CBP60g protein degradation was completely abolished (Figure 5c,d). These data indicate that RipAW directly affects the stability of CBP60g, presumably via its E3 ligase activity.

We then hypothesised that RipAW may associate with CBP60g for degradation. To avoid the possibility that RipAW‐triggered cell death on *N. benthamiana* might affect measurement of their interaction (Niu et al. 2022; Ouyang et al. 2023), RipAW was substituted with RipAW (C177S) to investigate the RipAW–CBP60g association. Given that CBP60g is a transcription factor that localises in the nucleus, we firstly investigated the subcellular localisation of GFP‐fused RipAW (C177S) in *N. benthamiana* leaf cells. To detect nuclei, mCherry fused to a nuclear localisation signal (NLS) was used (Figure S5). As previously shown, a fraction of RipAW (C177S)‐GFP localised at the plasma membrane (Sun et al. 2023) (Figure S5). However, we also detected strong RipAW (C177S)‐GFP fluorescence in the nucleus, overlapping with the nuclear mCherry signal (Figure S5). These data indicate that RipAW exhibits dual plasma membrane/nucleus localisation.

Next, we tested whether RipAW interacts with CBP60g using a split‐luciferase system, bimolecular fluorescence complementation (BiFC), and co‐immunoprecipitation (CoIP) assays. The interaction between XLG2 and BIK1, two important components of the PTI pathway in *Arabidopsis*, was used as a positive control, while the *Arabidopsis* transcription factor MYB34 was used as a negative control in the split‐luciferase assay (Celenza et al. 2005; Wang et al. 2021). Co‐expressing *RipAW (C177S)‐cLUC* with *CBP60g‐nLUC* generated strong luminescence, which did not occur when RipAW (C177S) was co‐expressed with MYB34 (Figure 5e). Similarly, the BiFC assay indicated RipAW association with CBP60g in the nucleus, but not with MYB34 (Figure 5f). Furthermore, the CoIP assay clearly showed that CBP60g associated with RipAW (C177S), but not with GFP‐FLAG (Figure 5g). These data provide several lines of evidence strongly supporting that RipAW interacts with and destabilises CBP60g. Expression of *SARD1* was too low in *N. benthamiana* to be detected; hence, the functional relationship between RipAW and SARD1 could not be investigated.

### 2.6 CBP60g and SARD1 Are Involved in Plant Resistance to RipAW‐Mediated *R. solanacearum* Multiplication in *Arabidopsis*, but Not in RipAW‐Mediated Root Architecture Changes

**FIGURE 6:**
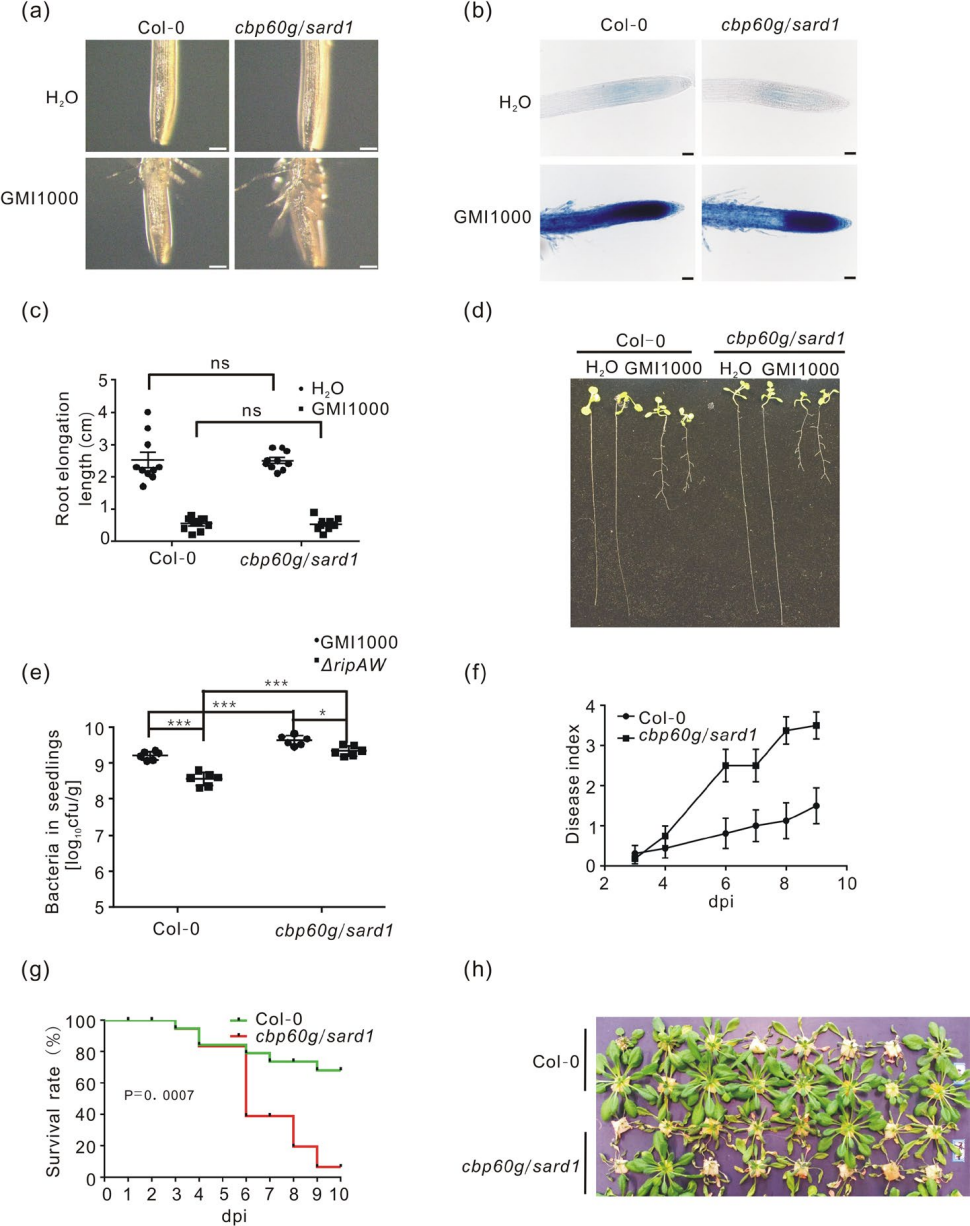
CBP60g and SARD1 are not involved in *Ralstonia solanacearum* ‐induced root architecture changes, but required for plant resistance to *R. solanacearum* . (a) Root hair formation on the root tip of *cbp60g/sard1* and wild type (WT) at 1 day post‐inoculation (dpi). Root hairs were photographed at 24 h post‐inoculation (hpi) with an Olympus microscope (*n* = 10, bar = 200 μm). (b) Cell death on roots of *cbp60g/sard1* and WT triggered by *R. solanacearum* at 2 dpi. Infected roots were stained with Evans blue and photographed using an Olympus microscope (*n* = 8, bar = 50 μm). (c) Root elongation length of *cbp60g/sard1* and WT plants infected with *R. solanacearum* at 4 dpi (*n* = 10). Statistical analysis was performed using two‐way ANOVA Sidak's test (ns, no significant difference). (d) Root growth of *cbp60g/sard1* and WT infected with *R. solanacearum* at 4 dpi (*n* = 10). (e) Bacterial growth of *cbp60g/sard1* mutant and WT seedlings. Bacteria were quantified in infected seedlings at 4 dpi (*n* = 12). Statistical analysis was performed using a two‐way ANOVA Sidak's test (* *p* < 0.05, ***p < 0.001). (f) Disease symptom development of *cbp60g/sard1* mutant and WT plants. Wilting symptoms of each plant was given a score from 0 (healthy plant) to 4 (completely dead plants) (*n* = 12). The experiment was repeated three times with similar results. (g) Survival analysis of (f). The data shown in (f) was transformed to survival analysis with the following criteria: Disease score lower than 2 was designated as ‘0’ while higher than 2 was designated as ‘1’. Statistical analysis was performed using log‐rank (Mantel‐Cox) test with *p* values shown in the graph. (h) Bacterial wilting symptoms on *cbp60g/sard1* mutant and WT plants. The digital image was taken at 10 dpi (*n* = 12). Six‐day‐old *cbp60g/sard1* mutant and WT seedlings were inoculated with *R. solanacearum* strains (OD_600_ = 0.01 for a–d, OD_600_ = 0.0001 for e). A 2 cm root fragment of 5‐week‐old *cbp60g/sard1* and WT plants grown in *Arabidopsis* nutrient solution was cut. The injured plants were transferred into a *R. solanacearum* GMI1000 suspension (OD_600_ = 0.1 for f–h). All experiments were done three times with similar results.

To determine the roles of CBP60g and SARD1 in plant defence against *R. solanacearum*, we first investigated the root phenotypes of the *cbp60g/sard1* double mutant after *R. solanacearum* infection compared to WT. Root hair formation, cell death on the root meristem and root growth inhibition were not altered in the *cbp60g/sard1* mutant (Figure 6a–d). These results indicate that CBP60g and SARD1 are not required for *R. solanacearum* ‐triggered root structure changes. To understand whether CBP60g and SARD1 are required for plant resistance to *R. solanacearum* , we analysed bacterial multiplication and disease symptoms in WT and *cbp60g/sard1* plants inoculated with *R. solanacearum* . Bacteria multiplied to higher levels in *cbp60g/sard1* compared to WT seedlings (Figure 6e). *cbp60g/sard1* mutant plants also showed earlier and faster appearance of wilting symptoms and a lower survival rate than WT plants (Figure 6f–h). Overall, these data indicate that CBP60g and SARD1 are important for plant basal defence to *R. solanacearum* .

**FIGURE 7:**
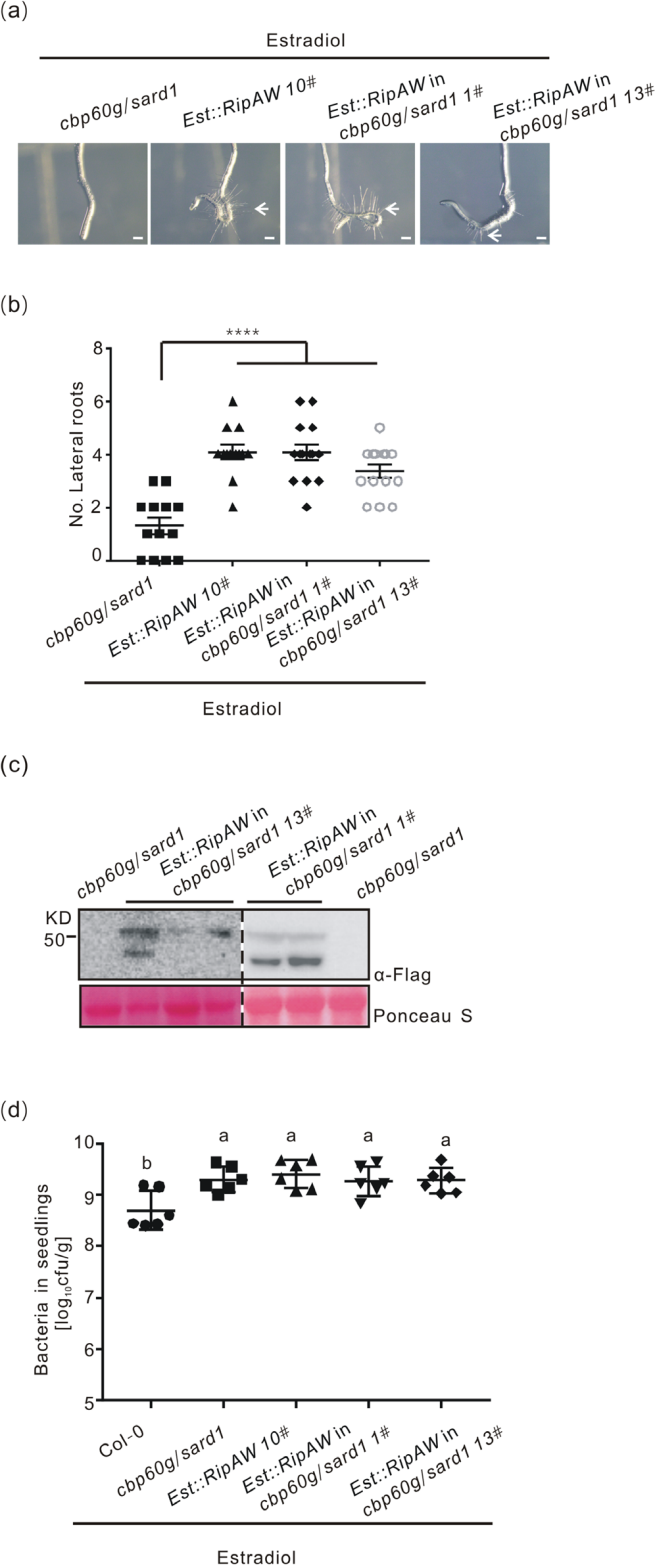
Lack of CBP60g and SARD1 does not alter RipAW‐mediated bacterial growth in plants. (a) RipAW‐induced root curvature on *cbp60g/sard1* seedlings. (b) RipAW‐induced lateral root formation on *cbp60g/sard1* seedlings. Six‐day‐old seedlings were transferred onto MS containing 5 μM β‐estradiol to induce *RipAW* expression and root structure changes were investigated. Root curvature phenotype was photographed at 2 days post‐β‐estradiol treatment (dpe) (*n* = 8, bar = 1 mm) and lateral roots were counted at 4 dpe (*n* = 10–13). The experiments were repeated three times with similar results. Arrow indicates root hairs. Statistical analysis was performed using one‐way ANOVA Dunnett's test (****p < 0.001). (c) Identification of *RipAW* expression in *cbp60g/sard1* seedling by western blot. (d) RipAW‐promoted bacterial growth in *cbp60g/sard1* seedlings. Six‐day‐old seedlings pretreated with 5 μM β‐estradiol were infected with *R. solanacearum* GMI1000. Bacterial colonisation was measured at 3 days post‐inoculation (*n* = 12). The experiment was repeated three times with similar results. Statistical analysis was performed using one‐way ANOVA Tukey's test (letters on columns indicates statistically significant differences).

To check whether RipAW virulence function in *R. solanacearum* proliferation is dependent on CBP60g/SARD1, *cbp60g/sard1* and WT seedlings were infected with the Δ*ripAW* mutant strain. Bacterial titres of the mutant strain in *cbp60g/sard1* seedlings were significantly higher than that in WT seedlings. More importantly, unlike what was observed in WT plants, the proliferation of the Δ*ripAW* strain was close to that of the WT strain in *cbp60g/sard1* seedlings (Figure 6e). This result suggests that mutation of CBP60g and SARD1 attenuates the negative effect on *R. solanacearum* proliferation in plants caused by lack of RipAW. To further verify the role of CBP60g and SARD1 in RipAW‐mediated pathogen virulence, we generated *cbp60g/sard1* mutant transgenic lines containing *Est::RipAW* and investigated whether RipAW‐mediated root morphology changes and bacterial growth were affected in *cbp60g/sard1* mutant. The expression of *RipAW* resulted in severe root curving and formation of lateral roots in both WT and *cbp60g/sard1* mutant plants (Figure 7a–c), indicating CBP60g and SARD1 are not involved in RipAW‐mediated root architecture changes. No differences in bacterial growth were observed between *cbp60g/sard1*, *Est::RipAW* 10# and *Est::RipAW cbp60g/sard1* transgenic lines, but all of them supported much more bacterial growth than WT plants (Figure 7d). These data indicate that the lack of CBP60g and SARD1 does not further increase RipAW‐mediated pathogen growth promotion, implying that RipAW increases bacterial proliferation through its negative regulation on CBP60g and SARD1.

## 3 Discussion

Upon detecting potential phytopathogens through cell surface receptors and intracellular receptors, plants rapidly activate diverse signalling pathways to counteract pathogen invasion. These various immune signalling pathways synergistically activate plant immunity to protect plants from invading agents. To subvert plant immunity, effectors from pathogens have been shown to target and inactivate key signalling nodes of these immune pathways to promote pathogen proliferation in plants (Bundalovic‐Torma et al. 2022). For example, multiple *P. syringae* effectors (AvrRPS2, AvrRPM1, AvrB, HopZ5) target RIN4, a PTI and ETI crosstalk node, through distinct enzymatic modifications (Bundalovic‐Torma et al. 2022). Recent studies have revealed that CBP60g and SARD1 constitute an important signalling hub of plant immunity, which participates in regulating SA biosynthesis, PTI, ETI and distal SAR (Peng et al. 2021; Sun et al. 2015). In this study, we demonstrated that the *R. solanacearum* effector RipAW regulates root architectures and promotes pathogen proliferation in plants. RNA‐seq analysis revealed that RipAW‐induced transcriptional reprogramming significantly upregulated CBP60g and SARD1. Loss‐of‐function mutants of *CBP60g* and *SARD1* exhibited increased pathogen colonisation and accelerated wilting symptoms. Furthermore, we found RipAW interacts with CBP60g, promoting its degradation in an E3 ligase‐dependent manner. Notably, RipAW‐induced changes in root architecture were unaffected in the *cbp60g/sard1* mutant plants. Collectively, our findings suggest that RipAW destabilises CBP60g and SARD1 to enhance bacterial virulence and manipulates root architectures to facilitate bacterial invasion in a distinct pathway. Interestingly, RipAW is not the only effector from pathogens to dampen plant immunity by disrupting CBP60g and SARD1 function. It has been reported that the fungal effector VdSCP41 associates with the C terminus of CBP60g and abolishes its transcriptional activation function, but does not affect CBP60g stability (Qin et al. 2018). Collectively, although both VdSCP41 and RipAW suppress CBP60g‐mediated plant immunity, they employ two distinct strategies: RipAW affects CBP60g stability, whereas VdSCP41 interrupts CBP60g transcriptional activity (Qin et al. 2018). These data together indicate pathogens independently evolve different strategies to disrupt CBP60g‐mediated immunity during long‐term plant–pathogen coevolution, supporting that CBP60g and SARD1 are another hotspot targeted by different pathogens to increase plant susceptibility, like RIN4 and BIK1.

Here we found that plants inducible expressing *RipAW* displayed reduced primary root elongation, root hair and lateral root formation (Figure 1). These phenotypes strongly resemble root phenotypes caused by *R. solanacearum* infection in plants (Lu et al. 2018; Zhao et al. 2019). In contrast to the root phenotypes of plants expressing *RipAW*, deletion of *RipAW* from the *R. solanacearum* genome alleviated altered root architecture changes (Figure 2). Abrogation of E3 ligase activity blocked all RipAW‐mediated root phenotype changes (Figure 3). Altogether, these data indicate RipAW is essential for root structure changes induced by *R. solanacearum* . The partial inhibition of *R. solanacearum* ‐triggered root phenotypes by RipAW deletion indicates that additional effectors may contribute to the observed root structure changes. This notion is supported by the fact that overexpression of *RipB* and *RipAC*, another two *R. solanacearum* effectors, remarkably inhibits primary root growth and promotes lateral root formation in *Arabidopsis* (Cao et al. 2022; Yu et al. 2024). Considering that lateral root emergence sites serve as key entry points for *R. solanacearum* (Vasse et al. 1995; Yu et al. 2024), it is plausible that the pathogen manipulates these structures to facilitate root invasion. A similar strategy has been observed in *P. syringae* pv. *tomato* (Pst), which promotes lateral root formation via activation of the ARF7‐dependent auxin pathway, thereby enhancing pathogen entry into the root (Kong et al. 2020). This suggests that manipulation of root architecture may be a common virulence strategy employed by various pathogens. Interestingly, auxin‐related GO terms were not enriched in RipAW‐triggered DEGs, implying that, unlike Pst, the auxin signalling pathway may not be required for RipAW‐induced lateral root formation. Notably, however, the auxin signalling pathway was strongly activated during *R. solanacearum* root infection (Zhao et al. 2019). This suggests that future studies should investigate whether *R. solanacearum* ‐triggered lateral root formation is dependent on the auxin signalling pathway. Additionally, RipAW does not appear to be involved in cell death induced by *R. solanacearum* , highlighting the need for further exploration into which effectors control root meristematic cell death and how this contributes to pathogen colonisation.

In addition to altering root architecture, the expression of RipAW in *Arabidopsis* also affects *R. solanacearum* growth (Figures 1, 2e and 3c). The *cbp60g/sard1* mutant plants supported significantly higher growth of *R. solanacearum* and exhibit earlier wilting symptoms and a lower survival rate than WT plants (Figure 6). Notably, the bacterial titres of Δ*ripAW* increased and approached those of the WT strain GMI1000 in the *cbp60g/sard1* mutant (Figure 6e), suggesting that the attenuated virulence of Δ*ripAW* can be partially rescued by the loss‐of‐function mutations of CBP60g and SARD1. Moreover, lack of CBP60g and SARD1 did not enhance RipAW‐mediated pathogen growth (Figure 7d). In line with these genetic data, RipAW was found to associate with CBP60g and induce its degradation. These data suggest that RipAW interferes with plant immunity by destabilising CBP60g and potentially SARD1.

In contrast to their importance in plant defence, mutations in CBP60g and SARD1 did not affect *R. solanacearum* ‐induced root phenotypes (Figure 6). Consistent with this, RipAW‐induced changes in root curvature and lateral root formation were unaffected in *cbp60g/sard1* mutants (Figure 7). These data suggest that CBP60g and SARD1 do not play a significant role in the root architecture changes induced by *R. solanacearum* and RipAW. One possible explanation is that RipAW may target other proteins or pathways in addition to CBP60g and SARD1 to modulate root morphology. This would not be surprising, because effectors typically target multiple host components (Bundalovic‐Torma et al. 2022). For example, *P. syringae* effector AvrPtoB targets several key components involved in PTI, ETI, SA and exocytosis to suppress plant immunity (Chen et al. 2017; Cheng et al. 2011; Gimenez‐Ibanez et al. 2009; Gohre et al. 2008; Wang et al. 2023, 2019). A similar phenomenon is also observed in *R. solanacearum* , where the effector RipAC targets SGT1 to suppress NLR‐mediated SGT1‐dependent plant immunity, while also associating with the E3 ligase PUB4 to destabilise BIK1 and suppress PTI (Yu et al. 2022, 2020). Recently, RipAC has also been shown to inhibit cellulose biosynthesis and promote lateral root formation, providing additional entry sites for the pathogen (Yu et al. 2024). Additionally, the *Arabidopsis* immune complex FLS2‐XLG2‐BIK1 has been reported to be another target of RipAW (Sun et al. 2023), which to some extent supports the possibility that RipAW triggers root phenotype through another target. In future, it will be good to know whether the immune complex affects *R. solanacearum* ‐induced root architecture changes, although *fls2* mutant seedlings do not show any growth defects (Gomez‐Gomez and Boller 2000).

Global analysis of RipAW‐induced transcriptional reprogramming revealed CBP60g and SARD1 were strongly induced, implying that SA‐dependent immunity is activated in RipAW‐expressing line because CBP60g and SARD1 can turn on the expression of SA biosynthetic genes (Zhang, Li, et al. 2010; Zhang, Xu, et al. 2010; Sun et al. 2015). However, either SA biosynthetic or signalling genes or SA content was unaffected by up‐regulated CBP60g and SARD1 expression (Figure 4, Figure S3, and Table 1). This puzzle can be explained by the data that RipAW associated with and destabilised CBP60g (Figure 5). Altogether, our data suggest that *R. solanacearum* suppresses SA‐mediated immunity by promoting RipAW‐induced degradation of CBP60g. Consistent with this, time‐resolved transcriptional profiling indicated that the expression of SA‐related biosynthesis genes and signalling genes was inactive during *R. solanacearum* infection (Hu et al. 2008; Zhao et al. 2019). In addition to RipAW, *R. solanacearum* employs other effectors to suppress SA‐mediated defence. RipAB interferes with the recruitment of TGA transcriptional activators to RNA Pol II, thus deactivating the SA signalling pathway (Qi et al. 2022), while RipAL activates jasmonic acid (JA) production to antagonise SA‐mediated immunity (Nakano and Mukaihara 2018). Avoidance of SA‐mediated immunity is a key strategy of *R. solanacearum* to invade plants. Different from RipAL and RipAB, RipAW also suppresses PTI by destabilising the critical immune complex FLS2‐XLG2‐BIK1 (Sun et al. 2023), which to some extent explains the fact that *cbp60g/sard1* mutation could not completely restore growth of Δ*ripAW* in plants (Figure 6e).

In this study, we have demonstrated that RipAW plays a critical role in *R. solanacearum* ‐induced root structural changes and pathogen colonisation. Although CBP60g and SARD1 are required for RipAW‐mediated pathogen colonisation, they are not involved in RipAW‐induced root architecture changes. This indicates RipAW facilitates *R. solanacearum* infection through two distinct mechanisms: one dependent on CBP60g and SARD1, and the other independent of these factors. Several important questions about the underlying mechanism of RipAW in *R. solanacearum* infection remain to be answered. First, why are *CBP60g* and *SARD1* expression activated in the *Est::RipAW* transgenic line, given that *R. solanacearum* destabilises them to successfully invade plants by RipAW? A similar observation was reported for the interaction between *P. syringae* and *Arabidopsis*, where virulent *P. syringae* strains successfully overcame the plant immunity system, but induced the strong expression of *CBP60g* and *SARD1* (Wang et al. 2009, 2011; Zhang, Xu, et al. 2010), the mechanism of which is still unclear. Notably, different from *P. syringae* strains, *R. solanacearum* invasion did not significantly increase the expression of *CBP60g* and *SARD1* (Hu et al. 2008; Zhao et al. 2019). Hence, it is possible that the plant senses the loss of CBP60g and SARD1 proteins resulting from the rapid large amount of RipAW appearance, then employs an unknown feedback mechanism to activate their expressions to maintain a certain level of CBP60g and SARD1 in plants, given their critical role in plant immunity (Sun et al. 2015). Nevertheless, this universal illogical phenomenon awaits elucidation. Secondly, if CBP60g and SARD1 are not involved in *R. solanacearum* ‐induced and RipAW‐triggered root structural changes, how does RipAW modulate root morphology? Thirdly, whether RipAW directly ubiquitinates CBP60g and whether the destabilisation of CBP60g is due to the ubiquitination of CBP60g by RipAW are two unresolved questions because we failed to purify CBP60g proteins out from *Escherichia coli* . Answering the above three questions will completely disclose RipAW virulence mechanism in plants, deepening our understanding of the interaction between the plant and *R. solanacearum* .

## 4 Experimental Procedures

### 4.1 Plant Materials and Microbial Strains

All *Arabidopsis* plants used in this study were from the ecotype Col‐0: WT, *cbp60g/sard1* double mutant, *RipAW* and its point mutation *RipAW (C177S)* transgenic lines, *Est::RipAW* in *cbp60g/sard1* double mutant. Seeds were sown onto plates containing solid MS without sugar (MS−) or MS with 20 g/L sucrose (MS2) medium and vertically grown in a controlled chamber under 23°C, short day photoperiod (8 h light and 16 h dark) and 70% humidity conditions. One week later, seedlings were ready for in vitro infection. *N. benthamiana* seeds were sown in soil and grown under the same conditions as *Arabidopsis*.

All strains in this study were grown at 28°C in liquid or solid rich B medium (1% peptone, 0.5% yeast extract and 0.5% casamino acids) supplemented with the appropriate antibiotics.

### 4.2 Plasmid and Transgenic Plants Generation

cDNA of *Arabidopsis* was used to amplify PCR fragments of *CBP60g* and *MYB34* with primers (Table S3), which were then cloned into pCambia1300‐HA (KpnI and SalI), pSPYCE(cYFP) (BamHI and XhoI) or pCambia1300‐nLUC (KpnI and SalI) for split‐luciferase, bimolecular fluorescence complementation (BiFC) and co‐immunoprecipitation (CoIP) assays, respectively (Zhou et al. 2018). Genomic DNA of *R. solanacearum* GMI1000 was used as a template for PCR amplification of *RipAW* (Rsp1475) fragment (primers detailed in the Table S3). PCR products were cloned into β‐estradiol‐inducible promoter vector pER8‐FLAG (XhoI and BstBI) (*Est::RipAW*), or pSPYNE (nYFP) (BamHI and XhoI) or pCambia1300‐cLUC (KpnI and SalI) (Zhou et al. 2018). *RipAW* and *RipAW (C177S)* in pCambia1300‐FLAG and pCambia6640‐GFP (BamHI and SalI) constructs have been described previously (Niu et al. 2022). These constructs were transformed into *Arabidopsis* ecotype Col‐0 using the *Agrobacterium tumefaciens* ‐mediated flower‐dipping method. The harvested T_1_ transgenic plants seeds were screened on MS2 plates containing hygromycin B to obtain the candidates of *Est::RipAW*, *35S::RipAW* and *35S::RipAW (C177S)* transgenic plants. *35S::RipAW* T_1_ transgenic plants showed growth defects and did not produce seeds. Homozygous T_3_ plants stably expressing *Est::RipAW* and *35S::RipAW (C177S)* were used for all assays.

### 4.3 Generation of the *R. solanacearum* Complemented Strain ( *C*Δ*ripAW* )

Genetic complementation of Δ*ripAW* mutant (Niu et al. 2022) was carried out with the Tn‐based site‐specific chromosomal integration system as reported (Choi et al. 2005; Zhang et al. 2021). *RipAW* containing its native promoter was integrated into the chromosome of Δ*ripAW* mutant 25 bp downstream of *GlmS* as a single copy. In brief, a DNA fragment containing the coding sequence of *RipAW* (Rsp1475 in GMI1000) and a 500 bp upstream region was PCR amplified with the primer pair Rsp1475A1B and Rsp1475B2H (Table S3) and subcloned into pUC18‐mini‐Tn*7*TGm. After validating the sequence, complementary *RipAW* was integrated into the chromosome of the Δ*ripAW* mutant strain and confirmed by colony PCR and quantitative PCR with primer pairs (Table S3) (Zhang et al. 2021).

### 4.4 *Ralstonia solanacearum* Infection Assays

For in vitro root morphology assay, 1‐week‐old *Arabidopsis* seedlings grown on MS− plates were inoculated with 10 μL of *R. solanacearum* solution with an optical density OD_600_ = 0.01. For root hair formation assay, around 10 infected seedlings were photographed with an Olympus microscope DP71 at 11.5× magnification. For cell death on root tip assay, eight infected roots at 2 dpi were stained with 0.05% Evans blue (Sigma) solution for half an hour at room temperature. Then roots were washed with water three times and photographed with an Olympus microscope BX53F at 20× magnification. For inhibition of root growth, root elongation from around 10 infected seedlings was measured with a ruler at 4–5 dpi. At the same time, the number of lateral roots was counted and photographed on infected seedlings. As for RipAW‐mediated root architecture changes, 1‐week‐old seedlings were transferred onto MS− plate containing 5 μM β‐estradiol and the root phenotypes were investigated.

The method for investigating root structure was further modified to measure RipAW‐mediated pathogen growth in vitro (Teixeira et al. 2021; Wang et al. 2024). Six‐day‐old seedlings were transferred onto 8% agar (Becton, Dickinson Co.) containing 5 μM β‐estradiol for 24 h, then a drop of bacterial solution at OD_600_ = 0.0001 was applied on the roots. Twelve seedlings per sample were divided into six groups, then weighed and homogenised in sterile distilled water at 3–4 dpi. Bacteria were quantified by colony counts on rich B medium plates.

For hydroponic infection, roots of 5‐ to 6‐week‐old plants grown in *Arabidopsis* nutrient solution (ANS) were cut and were transferred to bacterial solution (OD_600_ = 0.1) resuspended with ANS and incubated in the infection chamber at 26°C and long day photoperiod (16 h light and 8 h dark) (Liu et al. 2016). Wilting disease symptoms were evaluated and given a score from ‘0’ (healthy) to ‘4’ (complete wilting) at the indicated time (Lu et al. 2018). To calculate survival rates, wilting disease index for each plant was transformed into binary data with the following criteria: disease index lower than ‘2’ was referred to as ‘0’, other disease index was referred to as ‘1’ at the indicated times (Yu et al. 2020). Twelve plants for each genotype were used in this study.

### 4.5 Sample Preparation for RNA Seq and Data Analysis

The roots from 1000 6‐day‐old *RipAW* transgenic seedlings and WT seedlings were collected at 2 days after 5 μM β‐estradiol treatment, quickly frozen in liquid nitrogen and subjected to RNA sequencing using an Illumina sequencing platform (Novogene). Data analysis was performed by Novogene as follows. Raw data (raw reads) of fastq format were first processed through in‐house perl scripts by removing reads containing adapter, poly‐N and low‐quality reads. Then Q20, Q30 and GC contents were calculated, an average of 42 million clean reads with Q30 > 90% were obtained per sample. Paired‐end reads were aligned to the *Arabidopsis* reference genome using Hisat2 v. 2.0.5, more than 93% of the clean reads were uniquely mapped to the *Arabidopsis* genome (Table S1). The mapped reads of each sample were assembled by StringTie (v. 1.3.3b) in a reference‐based approach. Counts v. 1.5.0‐p3 was used to count the number of reads mapped to each gene. FPKM of each gene were calculated based on the length of the gene and read counts mapped to this gene. Finally, differential expression analysis of two conditions/groups (two biological replicates per condition) was performed using the DESeq2 R package. The resulting *p*‐values were adjusted using the Benjamini and Hochberg's approach for controlling the false discovery rate (FDR). Genes with an adjusted *p*‐value < 0.05 and fold change (FC) −0.5 > log_2_ FC or log_2_ FC > 0.5 were assigned as DEGs. Raw data for all RNA‐seq experiments will be available upon publication in the Sequence Read Archive under the BioProject accession code PRJNA985900.

### 4.6 GO Analysis

The up‐ and down‐regulated genes were loaded into the agriGO v. 2.0 online tool and GO analysis was performed using default settings (Tian et al. 2017). The top enriched GO terms with FDR < 0.05 were selected and presented.

### 4.7 RT‐qPCR

Seven‐day‐old *Est::RipAW* transgenic seedlings and WT seedlings were treated with ethanol or 5 μM β‐estradiol for 48 h. Total RNA from around 600 roots was extracted with the Gene JET Plant RNA Purification kit (Thermo Scientific) following the manufacturer's instructions. One microgram of total RNA served as template for synthesising first‐strand cDNA with All‐in‐One First‐Strand Synthesis SuperMix kit (TRANSGEN). One microlitre of the five‐fold diluted cDNA and primers were directly added into the SYBR Green mix (Gene Star). qPCRs were run on an iQ7 real‐time cycler machine (Life Technologies). Gene expression was quantified and normalised to *AtActin2* using the 2^−ΔΔ*C*t^ method with gene‐specific primers (Table S3).

### 4.8 SA Measurement

One milligram of root tissue from 4‐week‐old plants grown in ANS solution was treated with 25 μM β‐estradiol for 48 h and collected for measuring free SA content. SA was extracted and measured by UPLC‐ESI‐MS/MS, which was performed by LuMingBio company (Shanghai, China).

### 4.9 *Agrobacterium*‐Mediated Transient Expression on *N. benthamiana*

*Agrobacterium tumefaciens* GV3101 carrying the indicated genes was cultured in liquid Luria Bertani (LB) medium at 28°C overnight, then collected by centrifugation and suspended with the inoculation buffer (10 mM MES pH 5.0, 10 mM MgCl_2_, 400 μM acetosyringone). The bacterial solutions were kept in the dark for 4 h. Then the leaves of 5‐week‐old *N*. *benthamiana* were infiltrated with *A. tumefaciens* at OD_600_ = 1 and covered with a transparent plastic membrane. The inoculated plants were kept in a chamber under 23°C and long‐day conditions with 70% humidity. CHX (10 μg/mL) was infiltrated into leaves at 1 dpi, and total proteins were extracted for testing CBP60g stability after 3 h. The samples for subcellular localisation, split‐luciferase, BiFC and CoIP assays were measured at 2 dpi.

### 4.10 Subcellular Localisation, Split‐Luciferase Assay and BiFC Assay

*Nicotiana benthamiana* leaves were infiltrated with *A. tumefaciens* carrying indicated constructs. At 2 dpi, subcellular localisation and BiFC were investigated using laser scanning confocal microscopy (Zeiss; LSM880).

For split‐luciferase assay, *N. benthamiana* leaves co‐expressing the indicated constructs were sprayed with the substrate 1 mM luciferin. The luciferase activity of leaves was monitored using an ultra‐sensitive CCD camera (Alliance Q9 Advanced) (Zhou et al. 2018; Chen et al. 2008).

### 4.11 Co‐IP Assay

Total proteins in leaves expressing the indicated genes were extracted with the GTEN buffer (25 mM Tris pH 7.5, 150 mM NaCl, 1 mM EDTA, 10% glycerol, 1× protease inhibitor cocktail [Sigma], 1% Nonidet P‐40). The supernatants were incubated with 20 μL anti‐FLAG agarose conjugated antibody (Sigma‐Aldrich) for 4 h on a rotator in a cold room. Then the beads were washed with the washing buffer (25 mM Tris pH 7.5, 150 mM NaCl, 1 mM EDTA, 10% glycerol) three times and resuspended with SDS loading buffer. Samples were then separated by SDS‐PAGE and RipAW and CBP60g were individually detected with anti‐FLAG or anti‐HA antibodies, respectively (Sigma‐Aldrich).

### 4.12 Statistical Analysis

Statistical analyses were performed using the Graphpad Prism v. 7.0 software. Data are presented as mean ± SE. Experiments were repeated at least three times. All statistical methods and the number of biological replicates are indicated in the figure legends.

## Author Contributions

**Yang Niu:** data curation, formal analysis. **Tao Cao:** data curation, formal analysis. **Hui Ma:** data curation, formal analysis. **Shouyang Fu:** data curation, formal analysis. **Huijuan Wang:** data curation, formal analysis. **Qichang Gong:** data curation, formal analysis. **Shengyang Cheng:** data curation, formal analysis. **Dongdong Wang:** data curation. **Qin Chen:** project administration. **Marc Valls:** writing – original draft, writing – review and editing. **Nuria S. Coll:** writing – original draft, writing – review and editing. **Xiang Wang:** data curation. **Cuizhu Zhao:** conceptualization, investigation, funding acquisition, visualization, project administration, writing – original draft, writing – review and editing. **Yue Chen:** conceptualization, investigation, supervision, project administration. **Haibin Lu:** writing – original draft, writing – review and editing, visualization, validation, investigation, conceptualization, project administration, supervision, funding acquisition, resources. **Jinxue Hu:** data curation. **Min Chen:** data curation. **Yong Zhang:** investigation, validation, supervision.

## Funding

This work was supported by the Natural Science Basic research program of Shaanxi Province (2024JC‐YBMS‐180 and 2024JC‐YBMS‐144), the Fundamental research funds of Northwest A&F University (Z1090322151) and the Program of Introducing Talents of Innovative Discipline to Universities (Project 111) from the State Administration of Foreign Experts Affairs (B18042).

## Conflicts of Interest

The authors declare no conflicts of interest.

## Supporting information


**Figure S1:** Plants constitutive‐expressing *RipAW* exhibit defects in growth. (a) The growth phenotypes of 10‐week‐old Col‐0 plants and 10‐week‐old *35S::RipAW* T1 transgenic lines. Lines 1# and 2# showed sever dwarf phenotype while line 3# is less dwarf. All of them did not produce seeds. (b) Identification of *RipAW* expression in *Est::RipAW* transgenic lines. Six‐day‐old seedlings were transferred on MS2 containing 5 μM β‐estradiol. At 48 hpe, total proteins from seedlings were extracted and performed immunoblot with anti‐flag antibody for detecting RipAW protein.

**Figure S2:** RipAW plays an important role in *R. solanacearum* invasion on adult plants. (a) Characterisation of the complemented strain *C*Δ*RipAW* genotype by PCR. (b) Detection of *RipAW* expression in *C*Δ*RipAW* strain by qPCR. Gene expression was quantified and normalised to 16S *rRNA* using the 2^−ΔΔCt^ method. (c) The growth of different *R. solanacearum* strains in rich medium. (d) RipAW is required for *R. solanacearum* colonisation using a hydroponic infection system (*n* = 6). A 2 cm tip fragment of roots from 5‐week‐old plants grown in Arabidopsis nutrient solution (ANS) solution was cut off. Plants were then transferred into suspensions of *R. solanacearum* of the indicated strains (OD_600_ = 0.1). Bacterial populations were measured in the aerial parts of the plants at 5 dpi. Statistical analysis was done with one‐way ANOVA Tukey's test (*p < 0.05). The experiment was performed twice with similar results.

**Figure S3:** Identification the expressions of *CBP60g* and *SARD1* in *Est::RipAW* transgenic line 16#. Genes expressions of *CBP60g* and *SARD1* in *Est::RipAW* transgenic 16# seedlings were measured by RT‐qPCR at 48 hpe. Gene expression was quantified and normalised to *AtActin2* using the 2^−ΔΔCt^ method. The experiment was performed three with similar results. Statistical analysis was done with one‐way ANOVA Sidka's test (**p < 0.01, ***p < 0.001).

**Figure S4:** RipAW specifically affects CBP60g stablility. (a) RipAW promoted CBP60g degradation when they were co‐expressed in *N. benthamiana*. (b) RipAW could not trigger GFP‐HA‐nLUC degradation when they were co‐expressed in *N. benthamiana*. The indicated genes were transiently expressed in *N. benthamiana* by agrobacterium‐mediated transformation. At 1 dpi, the agro‐infiltrated leaves were treated with CHX for 4 h. The samples were collected and the indicated proteins were tested by western blot.

**Figure S5:** Subcellular localisation of RipAW (C177S). RipAW (C177S)‐GFP was transiently expressed on *N. benthamiana* by agroinfiltration. At 2dpi, subcellular localisation of RipAW (C177S)‐GFP were digitally photographed by a laser‐scanning confocal microscope (Zeiss LSM880), mCherry carrying with NLS was used to visualise nucleus, bar = 50 μm. The proteins were detected by western blot with anti‐GFP antibody.

**Data S1:** Differentially expressed genes in roots of *RipAW* transgenic plants and WT plants.

**Table S1:** Overview of RNA‐seq data quality.

**Table S2:** The expression of SA biosynthesis and signalling pathway genes in RNA‐seq data.

**Table S3:** Primers used in this study.

## Data Availability

The data that support the findings of this study are available from the corresponding author upon reasonable request.
