# Supplementary figures and images for: The Effector RipAW Enhances Ralstonia solanacearum Invasion in Arabidopsis via CBP60g/SARD1‐Dependent and ‐Independent Pathways
Source: Mol Plant Pathol. 2026 Jan 21;27(1):e70207. doi: 10.1111/mpp.70207 (PMC12824416; doi:10.1111/mpp.70207)

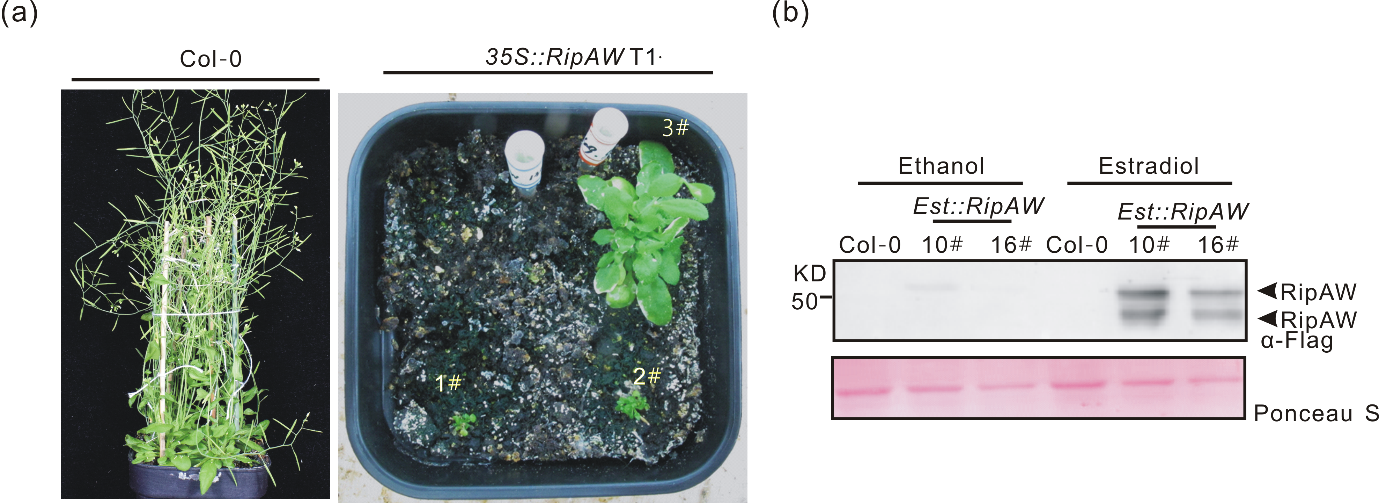


Figure S1

Supplement: Supplementary file 1 — Figure S1: Plants constitutive‐expressing RipAW exhibit defects in growth. (a) The growth phenotypes of 10‐week‐old Col‐0 plants and 10‐week‐old 35S::RipAW T1 transgenic lines. Lines 1# and 2# showed sever dwarf phenotype while line 3# is less dwarf. All of them did not produce seeds. (b) Identification of RipAW expression in Est::RipAW transgenic lines. Six‐day‐old seedlings were transferred on MS2 containing 5 μM β‐estradiol. At 48 hpe, total proteins from seedlings were extracted and performed immunoblot with anti‐flag antibody for detecting RipAW protein. [file MPP-27-e70207-s006.docx]

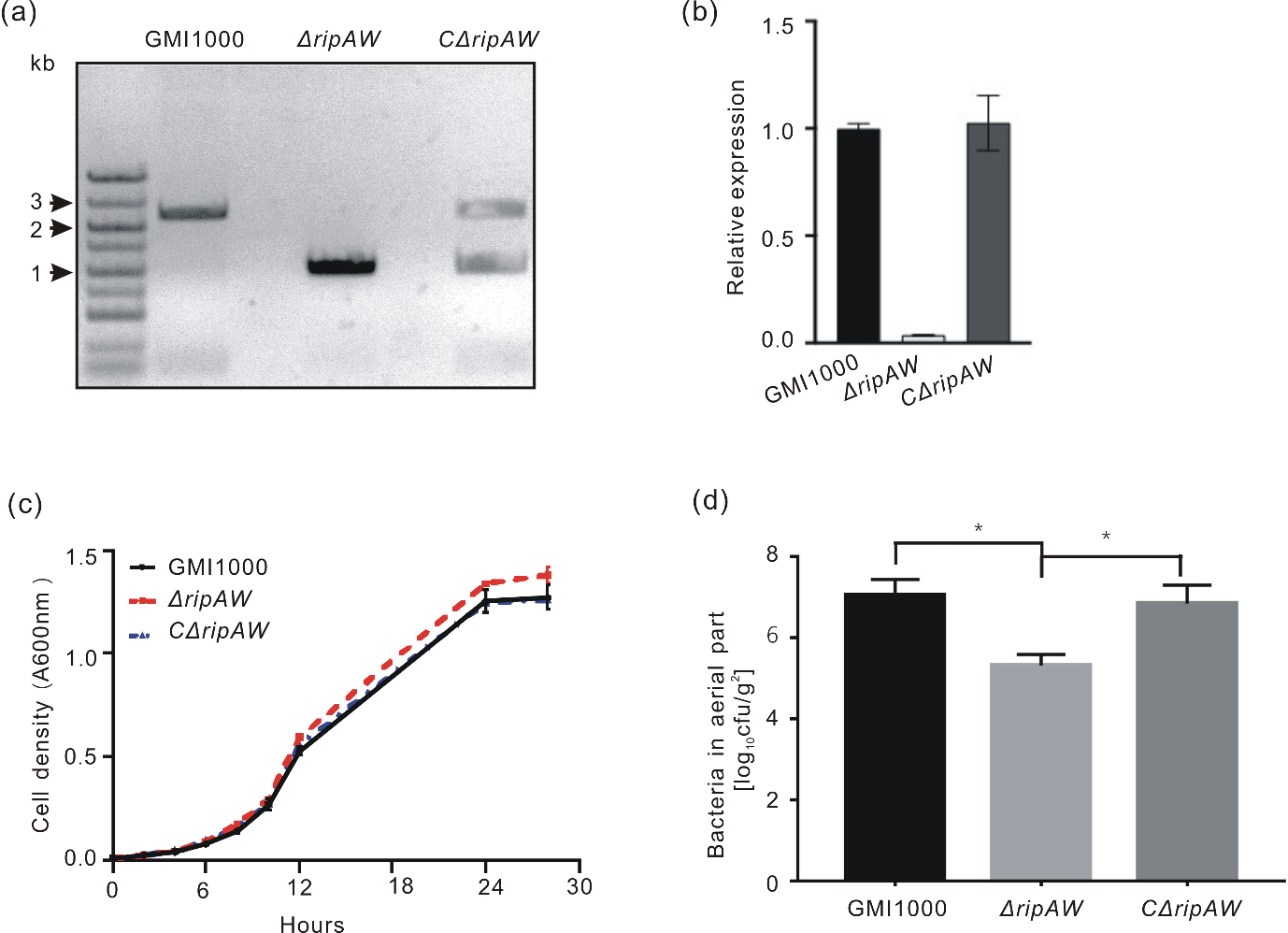


Figure S2

Supplement: Supplementary file 2 — Figure S2: RipAW plays an important role in R. solanacearum invasion on adult plants. (a) Characterisation of the complemented strain CΔRipAW genotype by PCR. (b) Detection of RipAW expression in CΔRipAW strain by qPCR. Gene expression was quantified and normalised to 16S rRNA using the 2−ΔΔCt method. (c) The growth of different R. solanacearum strains in rich medium. (d) RipAW is required for R. solanacearum colonisation using a hydroponic infection system (n = 6). A 2 cm tip fragment of roots from 5‐week‐old plants grown in Arabidopsis nutrient solution (ANS) solution was cut off. Plants were then transferred into suspensions of R. solanacearum of the indicated strains (OD600 = 0.1). Bacterial populations were measured in the aerial parts of the plants at 5 dpi. Statistical analysis was done with one‐way ANOVA Tukey's test (*p < 0.05). The experiment was performed twice with similar results. [file MPP-27-e70207-s001.docx]

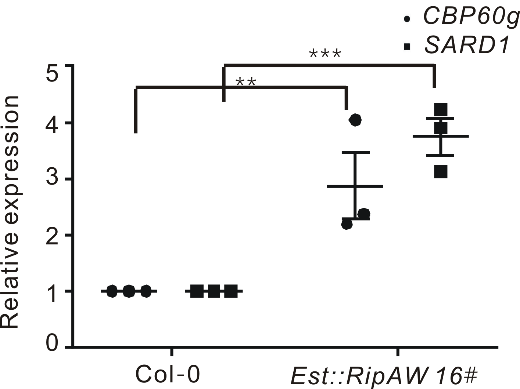


Figure S3

Supplement: Supplementary file 3 — Figure S3: Identification the expressions of CBP60g and SARD1 in Est::RipAW transgenic line 16#. Genes expressions of CBP60g and SARD1 in Est::RipAW transgenic 16# seedlings were measured by RT‐qPCR at 48 hpe. Gene expression was quantified and normalised to AtActin2 using the 2−ΔΔCt method. The experiment was performed three with similar results. Statistical analysis was done with one‐way ANOVA Sidka's test (**p < 0.01, ***p < 0.001). [file MPP-27-e70207-s009.docx]

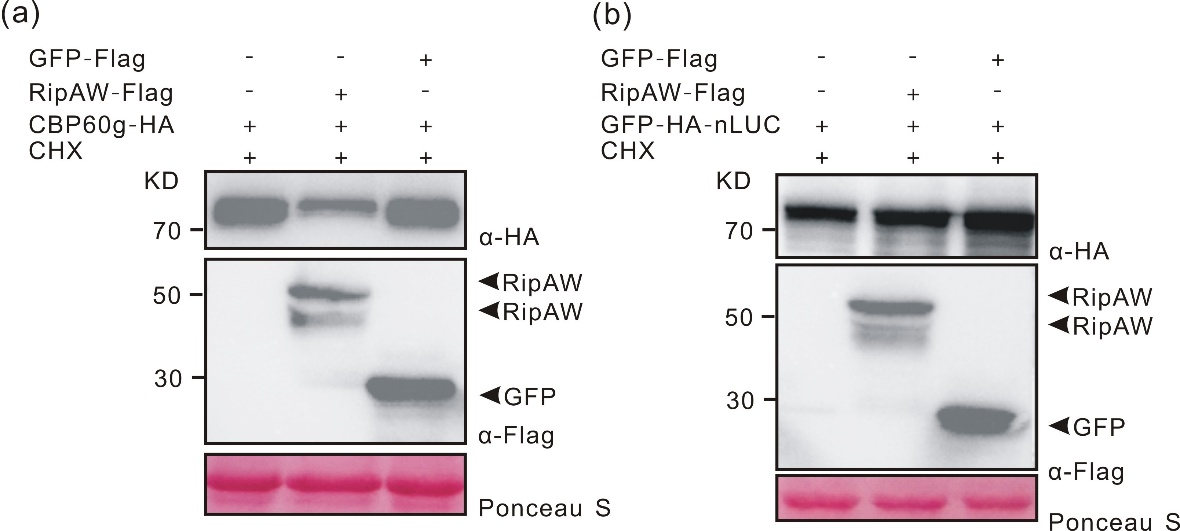


Figure S4

Supplement: Supplementary file 4 — Figure S4: RipAW specifically affects CBP60g stablility. (a) RipAW promoted CBP60g degradation when they were co‐expressed in N. benthamiana. (b) RipAW could not trigger GFP‐HA‐nLUC degradation when they were co‐expressed in N. benthamiana. The indicated genes were transiently expressed in N. benthamiana by agrobacterium‐mediated transformation. At 1 dpi, the agro‐infiltrated leaves were treated with CHX for 4 h. The samples were collected and the indicated proteins were tested by western blot. [file MPP-27-e70207-s003.docx]

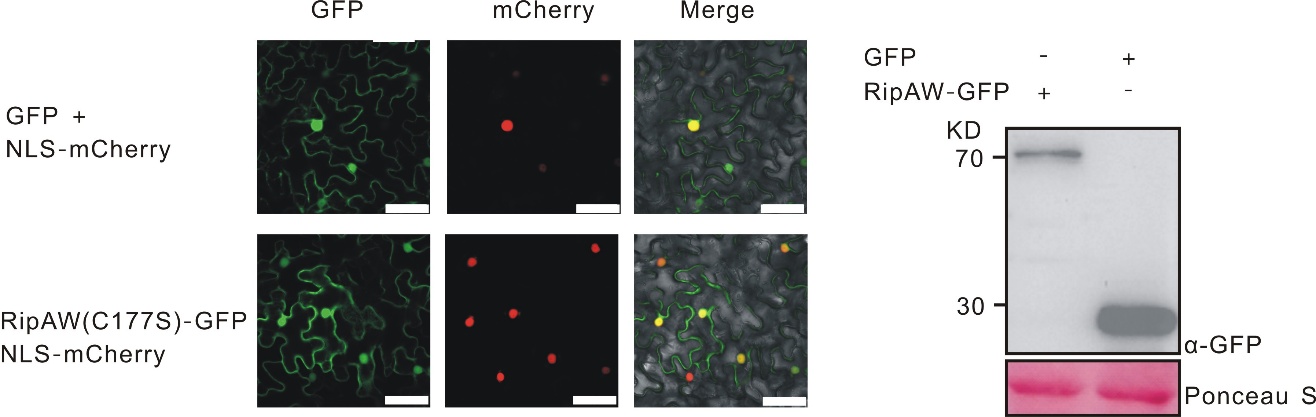


Figure S5

Supplement: Supplementary file 5 — Figure S5: Subcellular localisation of RipAW (C177S). RipAW (C177S)‐GFP was transiently expressed on N. benthamiana by agroinfiltration. At 2dpi, subcellular localisation of RipAW (C177S)‐GFP were digitally photographed by a laser‐scanning confocal microscope (Zeiss LSM880), mCherry carrying with NLS was used to visualise nucleus, bar = 50 μm. The proteins were detected by western blot with anti‐GFP antibody. [file MPP-27-e70207-s007.docx]
